# Icmt inhibition exerts anti-angiogenic and anti-hyperpermeability activities impeding malignant pleural effusion

**DOI:** 10.18632/oncotarget.7912

**Published:** 2016-03-04

**Authors:** Sophia Magkouta, Apostolos Pappas, Charalampos Moschos, Maria-Eleni Vazakidou, Katherina Psarra, Ioannis Kalomenidis

**Affiliations:** ^1^ “Marianthi Simou Laboratory”, 1st Department of Critical Care and Pulmonary Medicine, National and Kapodistrian University of Athens, School of Medicine, Evangelismos Hospital, Athens, Greece; ^2^ Department of Immunology - Histocompatibility, Evangelismos Hospital, Athens, Greece

**Keywords:** Icmt, malignant pleural effusion, adenocarcinoma, mesothelioma, GTPases

## Abstract

Small GTPases are pivotal regulators of several aspects of tumor progression. Their implication in angiogenesis, vascular permeability and tumor-associated inflammatory responses is relevant to the pathobiology of Malignant Pleural Effusion (MPE). Inhibition of isoprenylcysteine carboxylmethyltransferase (Icmt) abrogates small GTPase activation. We therefore hypothesized that cysmethynil, an Icmt inhibitor would limit pleural fluid accumulation in two models, a lung-adenocarcinoma and a mesothelioma-induced MPE. Cysmethynil significantly reduced MPE volume in both models and tumor burden in the adenocarcinoma model. It inhibited pleural vascular permeability and tumor angiogenesis *in vivo* and reduced endothelial cell proliferation, migration and tube formation *in vitro*. Cysmethynil also promoted M1 anti-tumor macrophage homing in the pleural space *in vivo*, and inhibited tumor-induced polarization of macrophages towards a M2 phenotype *in vitro*. In addition, the inhibitor promoted adenocarcinoma cell apoptosis *in vivo*. Inhibition of small GTPase might thus represent a valuable strategy for pharmacotherapy of MPE.

## INTRODUCTION

Malignant pleural effusion (MPE) i.e. the accumulation of fluid in the pleural cavity resulting from tumor cell infiltration of the pleura, is a common manifestation of a wide range of malignancies and is related to short life expectancy and significant morbidity [1, 2]. Current therapies for MPE are merely palliative, associated with side effects or discomfort and benefit only selected patients [3, 4]. Devising novel, safe and effective treatment modalities is therefore urgently needed.

Small GTPases are G-proteins involved in the transduction of signals that promote epithelial cell transformation and cancer cell proliferation, survival, migration and invasion thus being critical to tumor initiation, progression and metastasis [5, 6]. Besides their role in tumor cell biology, they orchestrate several host-related tumor-promoting phenomena such as angiogenesis [7–9], vascular permeability [10, 11], and tumor-associated inflammatory responses [12]. Interestingly, all these aforementioned biological procedures constitute the pathogenetic basis of MPE [13]. We therefore assumed that the inhibition of small GTPases would limit MPE accumulation. Inhibition of the Icmt enzyme prevents post-translational modification of GTPases that is required for their activation and this might be the most effective way to block GTPase activity [14]. In the studies presented here, we aimed at investigating the effects of the Icmt inhibitor cysmethynil [15] in experimental MPE [16, 17] and explore the underlying mechanisms. We hypothesized that cysmethynil would block MPE accumulation by inhibiting angiogenesis and pleural vascular permeability and by modulating tumor-initiated host immune response.

## RESULTS

### Cysmethynil blocks malignant pleural effusion accumulation in both models and reduces tumor burden in the lung-adenocarcinoma model

**Figure 1:**
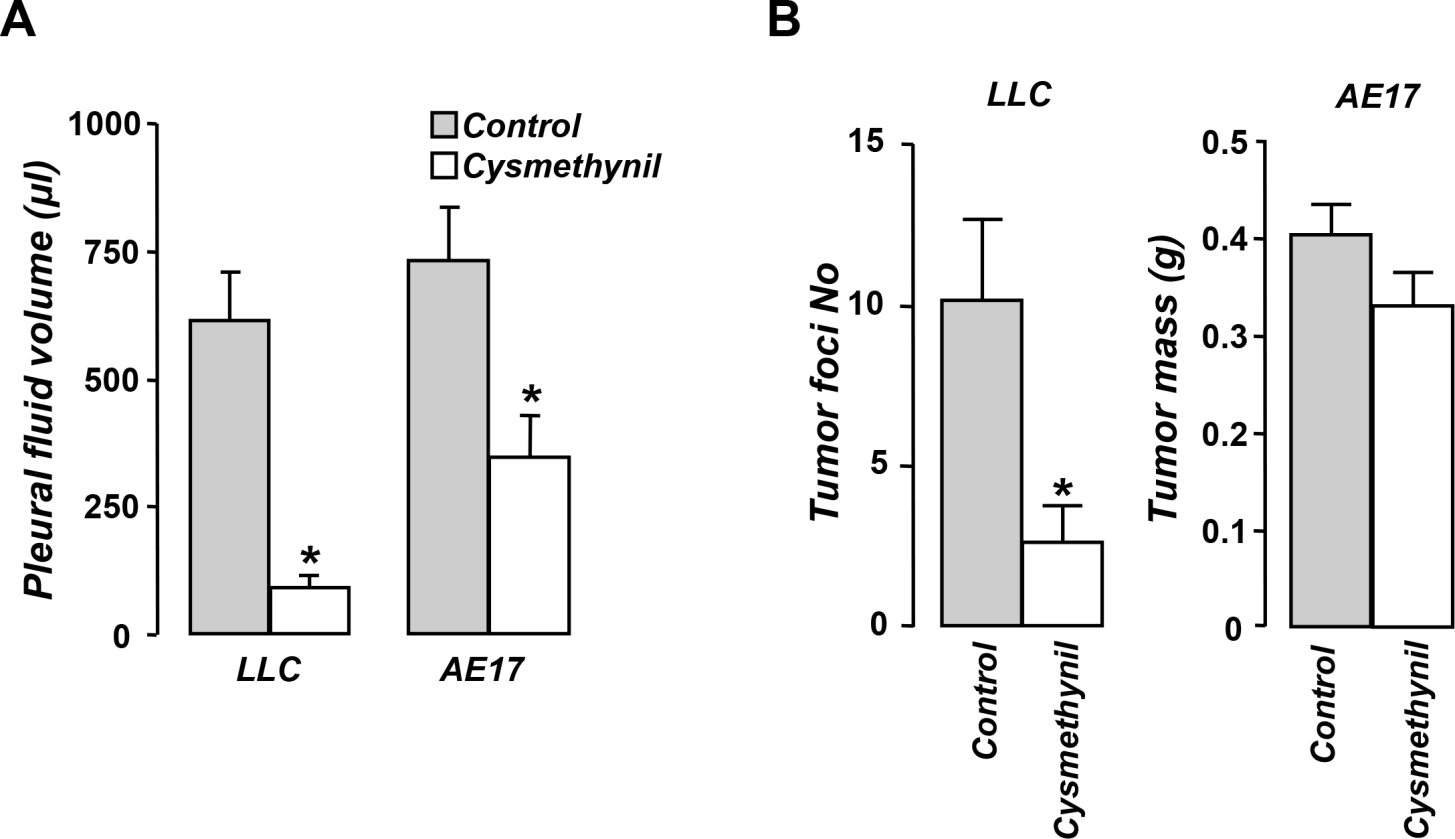
Cysmethynil inhibits pleural fluid accumulation in adenocarcinoma and mesothelioma-induced MPE and reduces adenocarcinoma pleural dissemination LLC or AE17 cells were intrapleurally injected in syngeneic mice. Mice were intraperitoneally administered with Cysmethynil 0.2 mg/g (body weight) or vehicle thrice per week. 11–14 days later, pleural fluid was retrieved and quantified (**A**). (**B**) Mesothelioma tumors were excised and weighed and adenocarcinoma lung implantations were counted under a stereoscope. Data presented as mean ± SEM, *n* = 6–11, *p < 0.05 compared to vehicle.

In order to examine whether cysmethynil affects MPE accumulation we used two syngeneic murine models: an adenocarcinoma and a mesothelioma-induced MPE. Cysmethynil significantly reduced pleural fluid volume in both models (Figure 1A). Even though cysmethynil reduced pleural fluid accumulation in both models, it impacted pleural tumor burden differently. While it significantly reduced the number of tumor foci in the adenocarcinoma model it did not affect tumor mass in the mesothelioma one (Figure 1B).

### Cysmethynil targets pleural vascular permeability and tumor angiogenesis

**Figure 2:**
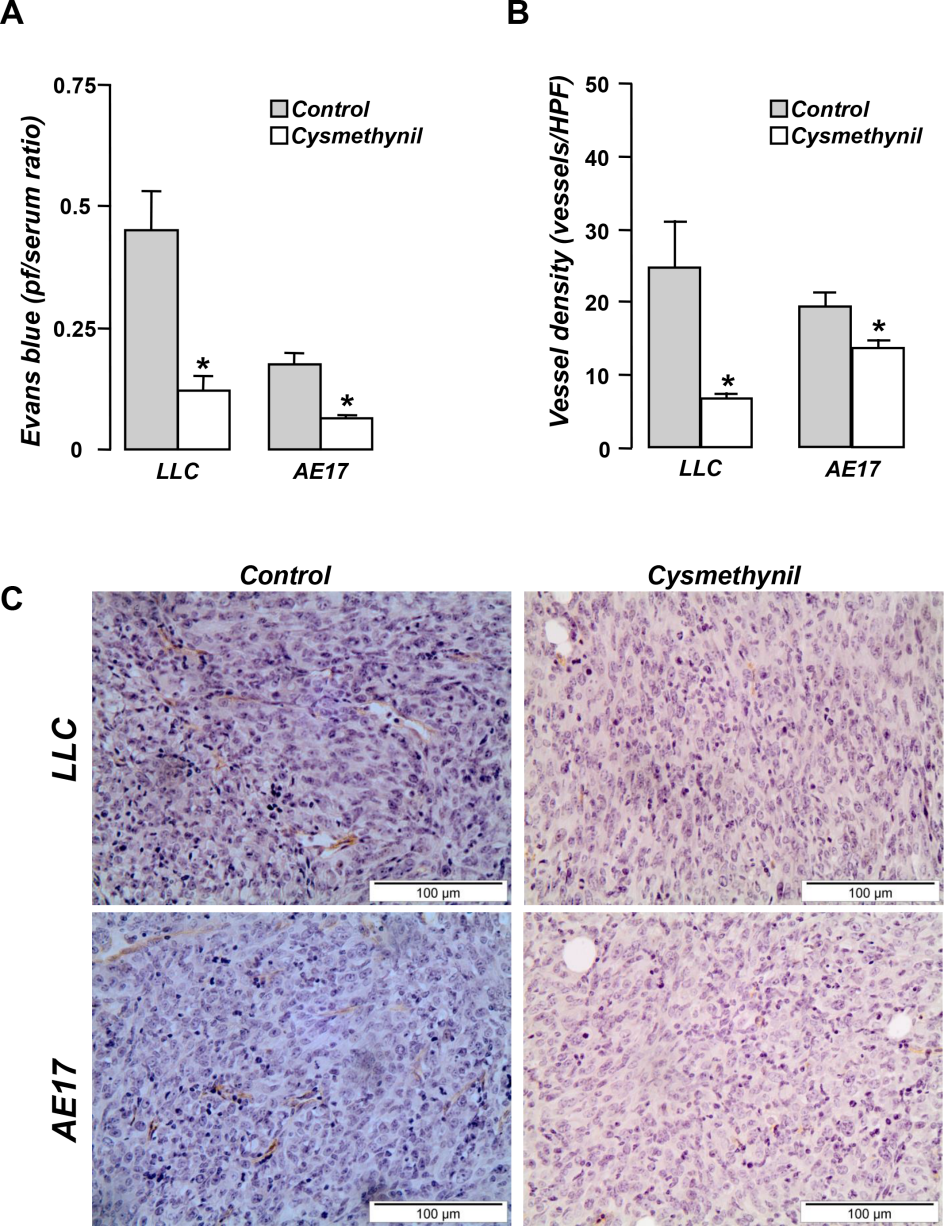
Cysmethynil inhibits pleural vascular hyper-permeability and tumor angiogenesis (**A**) MPE-bearing mice received 800 μg of Evans blue i.v. and sacrificed 1 h later. Vascular permeability was evaluated according to the pleural fluid (pf)/serum Evans blue concentration. (**B**) Tumor tissue sections were stained with endothelial marker CD31 and vascular density was assessed. (**C**) Representative photographs of CD-31 stained tumor tissue sections at 400x. HPF: High Power Field. Data presented as mean ± SEM, *n* = 5–7. *p < 0.05 compared to vehicle.

We subsequently investigated the mechanisms of the effusion-limiting effects of cysmethynil. We thus examined whether cysmethynil affects fundamental components of MPE pathobiology: vascular permeability, angiogenesis and tumor associated inflammatory response [12]. We demonstrated that the inhibitor significantly reduced pleural vascular permeability (Figure 2A) and tumor angiogenesis in both models (Figure 2B and 2C).

### Cysmethynil limits endothelial cell growth, migration and tube formation

**Figure 3:**
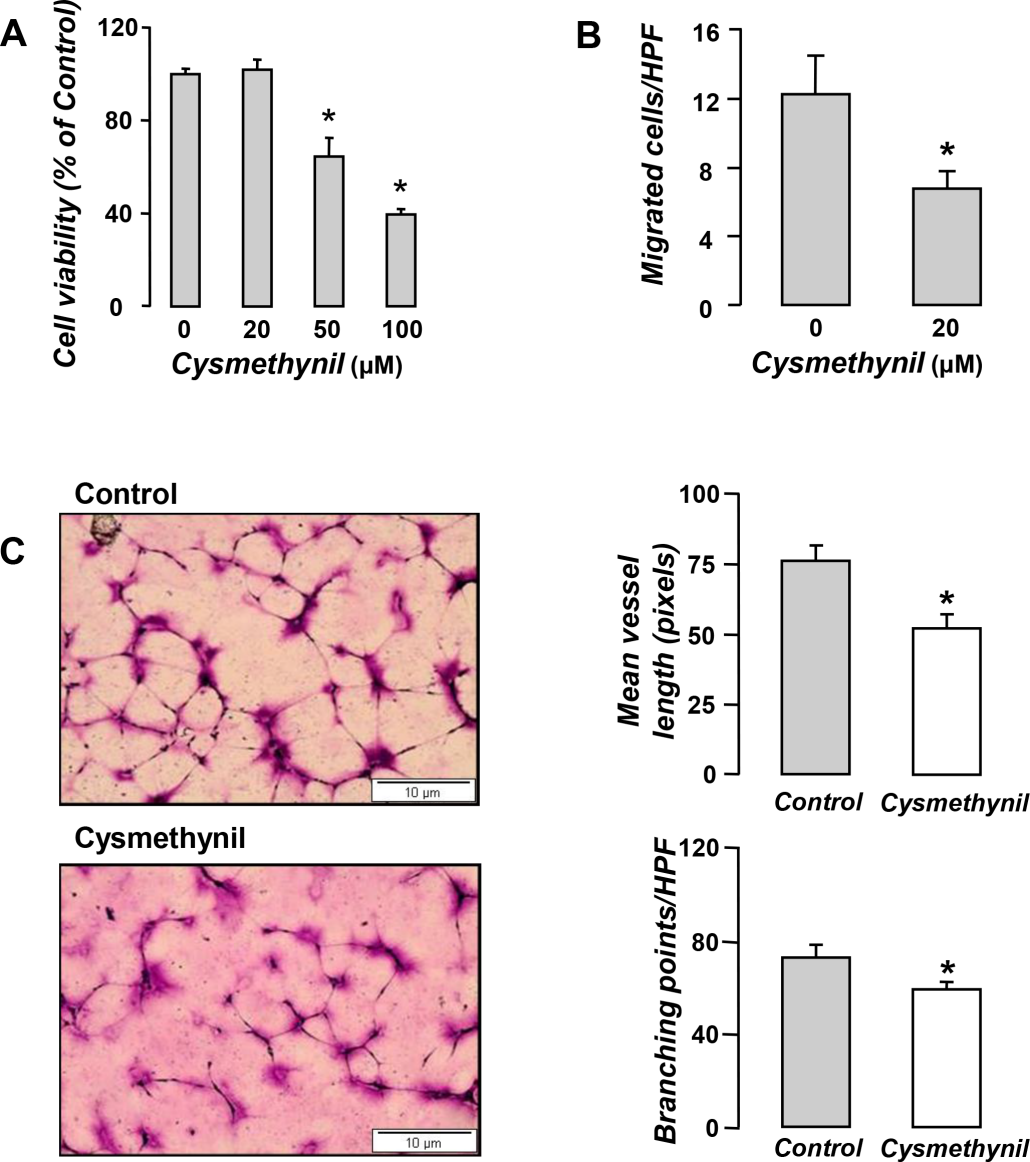
Anti-angiogenic properties of Cysmethynil *in vitro* (**A**) HUVECs were treated with vehicle or cysmethynil (20–100 μM) and 24 h later cell viability was evaluated by MTS. (**B**) For migratory potential evaluation, HUVECs were loaded onto Transwell inserts in the presence of cysmethynil or vehicle. Lower chambers were filled with full medium. 6 hours later, migrated cells were fixed, stained and counted under a microscope. (**C**) Serum starved HUVECs were loaded onto Matrigel coated wells and incubated overnight in the presence of cysmethynil (0.5–20 μM) or vehicle. (Left) Representative pictures of the tubular network. (Right) Results of image analysis for network length and branching points using ImageJ (NIH, Bethesda, MD). HPF: High Power Field. Data presented as mean ± SEM, *n* = 6–9 *p < 0.05 compared to vehicle.

In order to elucidate the mechanisms of the *in vivo* anti-angiogenic effects of the inhibitor we asked whether cysmethynil directly affects critical endothelial cell functions including growth, migration and *de novo* vessel formation in Matrigel. We found that cysmethynil significantly inhibited endothelial cell viability (Figure 3A) and suppressed their migration by almost 50% (Figure 3B). We then used the matrigel-based system to investigate the ability of endothelial cells to form two-dimensional, capillary-like tubes *de novo* (Figure 3C). We thus measured the total vessel length and the number of branching points (signaling the size and complexity/maturity of the network) in the tube networks. Cysmethynil-treated endothelial cells formed shorter and less complicated vessel networks than the vehicle-treated ones (Figure 3C).

To further assess any effect of cysmethynil in the angiogenic potential of tumor cells we subsequently measured VEGF levels in the tumor lysates, pleural fluids and tumor cell supernatants. No significant difference in VEGF levels was measured in tumors, fluid or cells exposed to the inhibitor compared to the control ones (data not shown) implying that the anti-angiogenic effects of cysmethynil should be more likely ascribed to its direct effects on endothelial cells.

### Cysmethynil promotes pleural space macrophage homing and their shift towards an M1-phenotype

**Figure 4:**
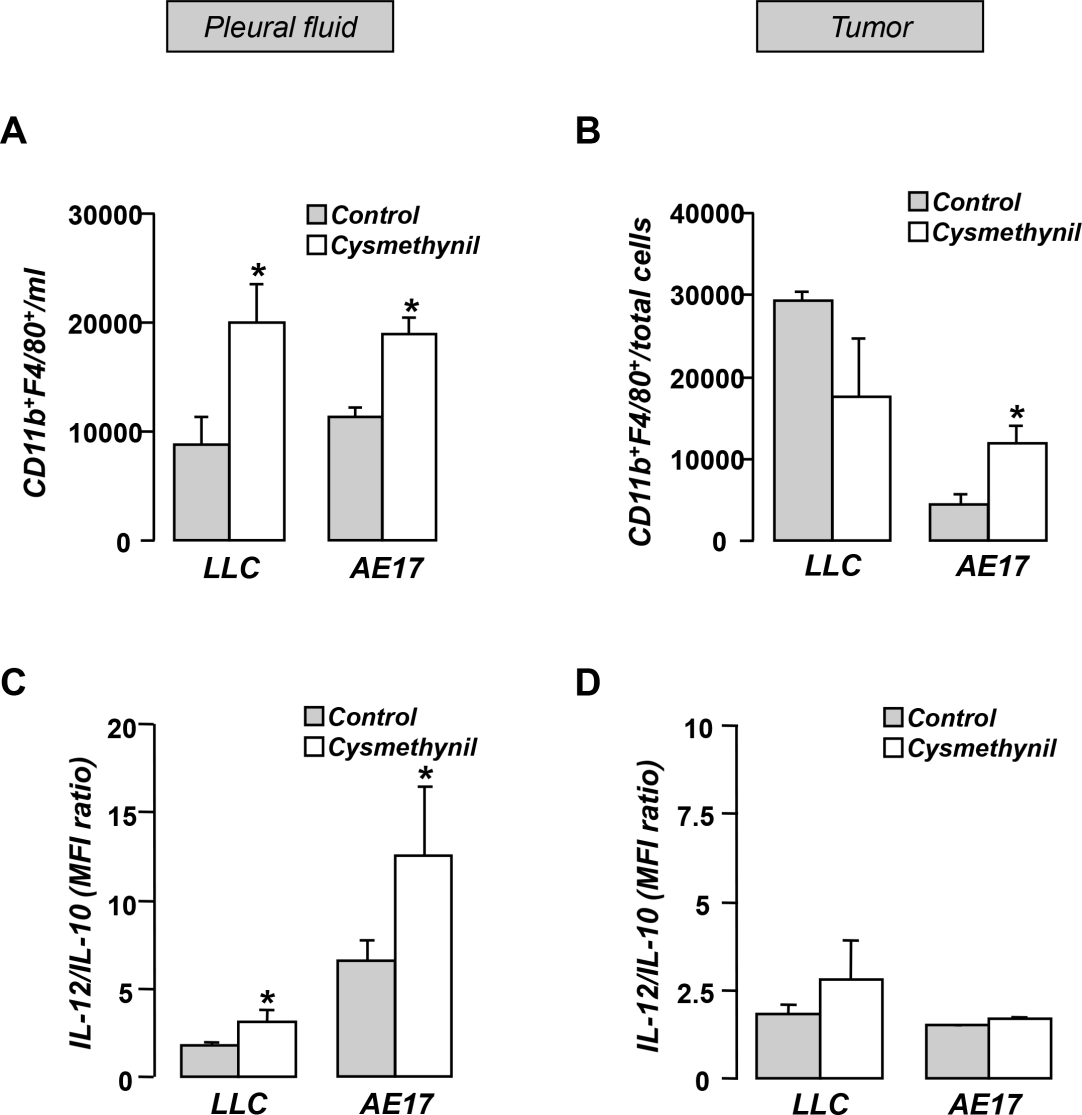
Pleural fluid (**A**) and tumors (**B**) of LLC or AE17 MPE-bearing animals were analyzed for the presence of CD11b+/F4/80+ macrophages. The population was further analyzed for the IL-12/IL-10 expression using flow cytometry (**C**, **D**). Data presented as mean ± SEM, *n* = 6–7 *p < 0.05 compared to vehicle.

Tumor associated inflammation plays a major role in malignant pleural effusion accumulation [13]. Although no significant difference was observed in total cellularity of pleural effusions among groups (data not shown), cysmethynil treated animals exhibited increased numbers of pleural space macrophages (Figure 4A). Moreover, analysis of the IL-12/IL-10 expression profile of the pleural macrophages demonstrated a significant shift towards an M1 anti-tumor phenotype in cysmethynil treated animals (Figure 4C). In tumors, the inhibitor-treated mesothelioma-bearing animals had higher numbers of macrophages compared to controls (Figure 4B), while no significant difference was observed in the adenocarcinoma model (Figure 4B). Finally, no significant effect in the intra-tumoral macrophage polarization was observed in both models (Figure 4D).

### Cysmethynil inhibits tumor-induced M2 macrophage polarization and enhances macrophage migration *in vitro*

**Figure 5:**
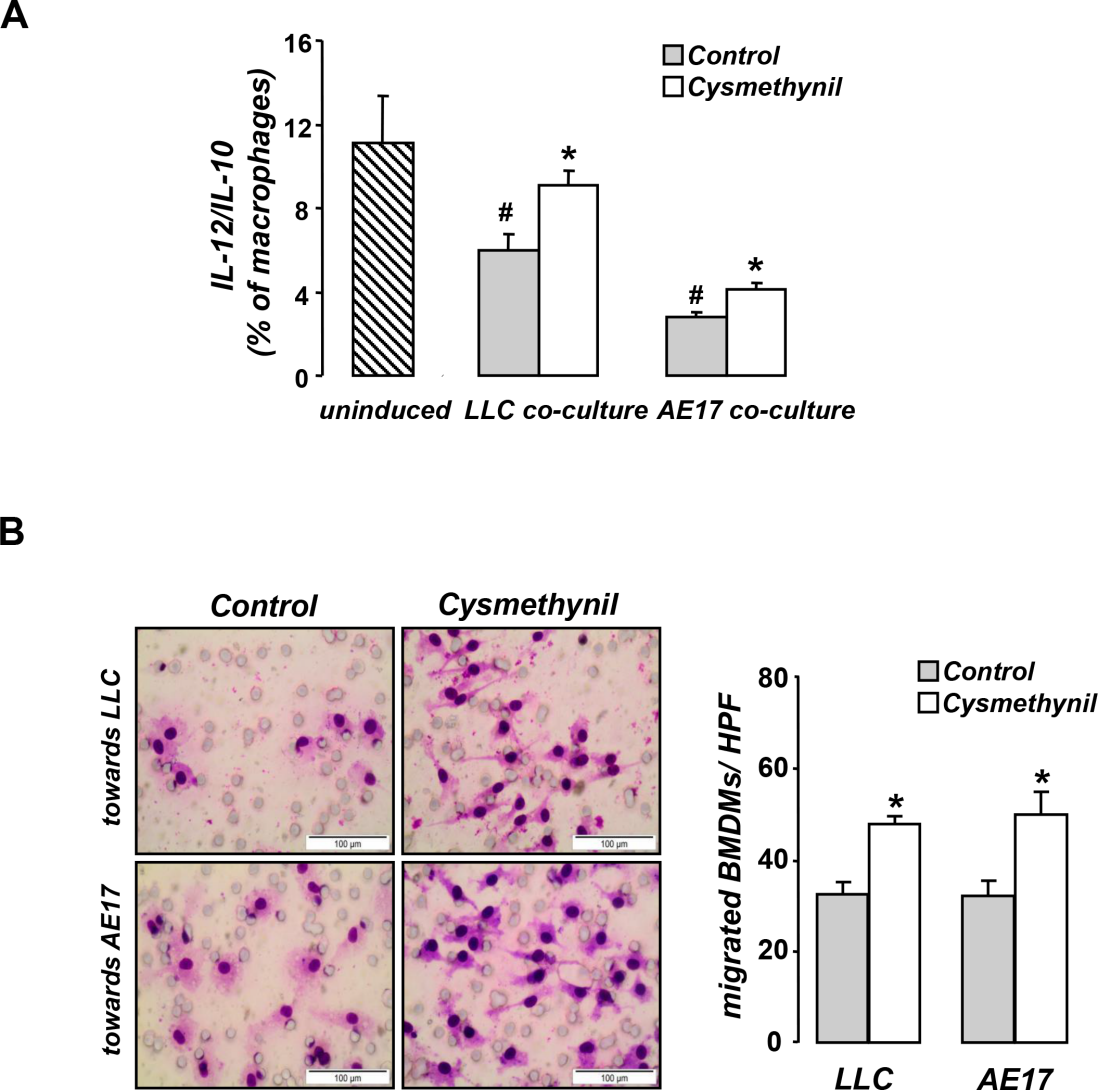
Cysmethynil inhibits tumor-driven M2 polarization (A) and migration (B) of macrophages *in vitro* (**A**) Macrophages were co-cultured with LLC or AE17 cells for 24 h and subsequently treated with vehicle or 20 μm Cysmethynil. 24 h later, macrophages were collected, stained for CD11b/F4/80/IL-12/IL-10 expression and analyzed by flow cytometry. (**B**) For migration studies, serum starved macrophages treated with vehicle or 20 μm Cysmethynil were loaded on Transwell inserts to migrate towards LLC or AE17 cells. (Left) Representative pictures of migrated cells. (Right) Results of migrated cell counts. HPF: High Power Field. Data presented as mean ± SEM, *n* = 6–7 *p < 0.05 compared to vehicle, #*p* < 0.05 compared to uninduced.

We then tested the effect of the inhibitor in macrophage polarization and migration *in vitro*. When co-cultured with tumor cells, macrophages were polarized towards an M2 phenotype and this alteration was partially reversed by cysmethynil (Figure 5A). In addition, cysmethynil promoted macrophage migration towards tumor cells (Figure 5B).

### Cysmethynil promotes adenocarcinoma cell apoptosis *in vivo*

**Figure 6:**
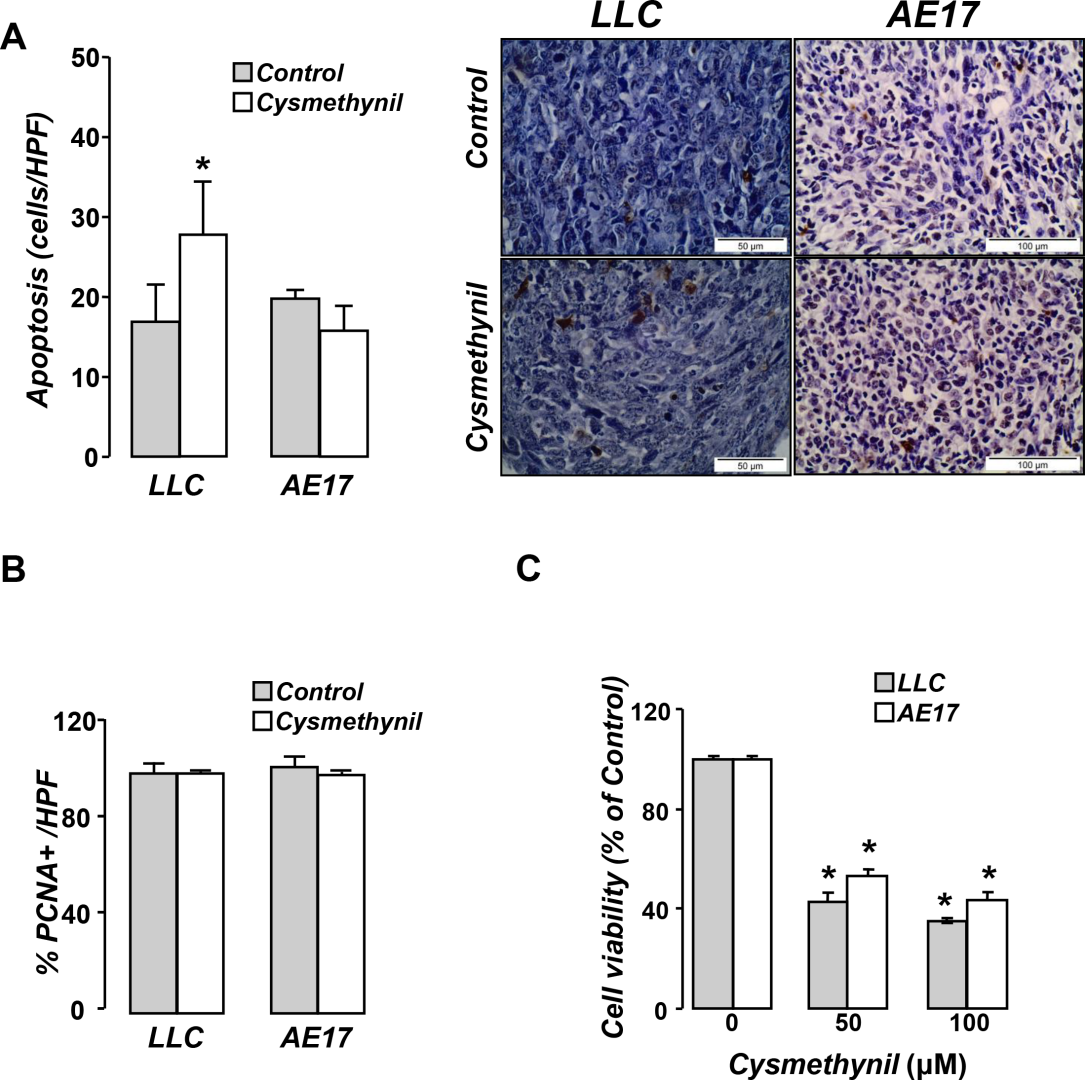
Cysmethynil induces tumor cell apoptosis *in vivo* and reduces tumor cell viability *in vitro* Τumor tissue sections from vehicle or cysmethynil treated animals were analyzed for apoptosis by TUNEL assay (**A**) while tumor cell proliferation rates were evaluated by PCNA staining (**B**). Data presented as mean±SEM, *n* = 7–10 *p < 0.05 from vehicle. HPF: High Power Field. (**C**) Tumor cells were treated with vehicle or cysmethynil (20–100 μM) and 24 h later cell viability was evaluated by MTS. Data presented as mean ± SEM, *n* = 5–7. *p < 0.05 compared to vehicle.

Pleural tumor tissues were subsequently analyzed for tumor cell apoptosis (Figure 6A) and proliferation rate (Figure 6B). Cysmethynil promoted tumor cell apoptosis in the adenocarcinoma but not in the mesothelioma model. Proliferation rate did not differ between groups in both models. Cysmethynil was also found to reduce tumor cell viability *in vitro* (Figure 6C).

## DISCUSSION

In the studies presented here we aimed at examining whether cysmethynil, a small GTPase inhibitor, possesses an MPE-limiting capacity, using two experimental MPE models (mesothelioma- and lung adenocarcinoma-induced MPE) established by our group. We demonstrated that cysmethynil: a. Profoundly reduced pleural fluid volume in both models and suppressed pleural adenocarcinoma tumor dissemination while mesothelioma tumor growth was not affected; b. Inhibited pleural vascular permeability and tumor angiogenesis *in vivo* and reduced endothelial cell proliferation, migration and tube formation *in vitro*; c. promoted pleural but not tumoral macrophage infiltration, forced pleural macrophages towards an M1 anti-tumor phenotype *in vivo*, and partially prevented a tumor-cell induced M2 polarization of macrophages *in vitro*; d. promoted adenocarcinoma but not mesothelioma cell apoptosis *in vivo*.

This is the first study to show that Icmt inhibition limits MPE. Previous studies have focused on the role of Icmt in tumor initiation and progression [15, 19, 23–26]. Notably, while cysmethynil reduced the size of MPEs in both models, it differentially affected tumor progression: it stimulated apoptosis *in vivo* and reduced the number of pleural tumor foci in the adenocarcinoma model, but did not affect mesothelioma cell survival *in vivo* or the size of mesothelioma tumors. While in the *in vitro* setting, adenocarcinoma cells were more sensitive to the inhibitor than mesothelioma ones, the magnitude of this difference is marginal. It is therefore unlikely that different sensitivity to the compound can explain the divergent *in vivo* responses. Since adenocarcinoma pleural growth is characterized by disseminated small foci [16] while mesothelioma forms massive tumors [17] it could be speculated that different pharmacokinetics may play a role in the observed different response *in vivo*. The observation that the MPE size was independent of the effects of the inhibitor in tumor burden, supports the assumption that the mechanisms determining the pleural fluid accumulation and the tumor growth are different. On the other hand, it should be emphasized that our findings should not be interpreted as evidence that mesotheliomas and lung adenocarcinomas in general would be expected to respond to the compound in a fashion similar to that of AE17 and LLC cells, respectively.

We here demonstrate for the first time, that Icmt, besides inhibiting tumor cell growth, may impact certain host-related hallmarks of cancer, i.e. vascular changes and tumor-associated immune response. The anti-angiogenic and the anti-hyperpermeability properties of the drug are probably central to its anti-MPE activity, since vascular changes represent sine qua non components of MPE pathogenesis [13]. Cysmethynil blocked *in vitro* endothelial cell proliferation, migration and tube formation, suggesting a direct effect of the inhibitor on endothelial cells. These vascular effects of the inhibitor should be possibly ascribed to its small GTPase-inhibitory properties. In fact, activation of endothelial cell RhoA, RhoG, Rac1 and cdc42 has been shown to stimulate several steps of the angiogenic process [8, 9] and endothelial cell RhoA mediates induction of vascular hyper-permeability [10, 11].

Another essential aspect of MPE pathobiology is the host immune/inflammatory response to pleural tumors [13]. We observed increased numbers of pleural space macrophages in the inhibitor-treated animals, a finding that comes in agreement with the previously observed accumulation of pan-Rho-deficient macrophages in the peritoneal cavity in a peritonitis model [27]. In addition, MPE macrophages were shifted towards an M1 phenotype in cysmethynil-treated animals compared to the control ones and, *in vitro*, the inhibitor suppressed tumor cell-driven M2 macrophage polarization. Even though the role of M2 macrophages in tumor progression has been long established [28], there is no direct experimental proof that they promote MPE. However, this is likely to be the case since they secrete pro-angiogenic and vascular-permeability-enhancing mediators which are implicated in MPE pathobiology [13]. Moreover, pleural fluid M2 abundance has been associated with worst prognosis in patients with MPE [29]. Surprisingly, while cysmethynil uniformly increased MPE macrophage numbers in both models, only mesothelioma tumors were found to be affected in this fashion. In addition, while the inhibitor induced M1-polarization of fluid macrophages, the phenotype of tumor macrophages was not affected. The reason of this discrepancy remains elusive. However, this anatomic compartment-dependent effect denotes that the effects of the inhibitor on macrophages might be substantially influenced by other factors released in specific *in vivo* micro-environments.

This proof-of-principle study supporting the notion that Icmt inhibitor may block MPE formation holds certain clinical implications. First, our findings may pave the road for clinical development of Icmt inhibitors [26] and clinical testing in patients with MPE. Second, the fact that the inhibitor targets host-related aspects of MPE pathobiology, may suggest that its activity is irrespective of specific genetic profile of the tumors that cause the effusion. Third, drugs targeting host-related cancer hallmarks might be less prone to the emergence of resistance, since host cells are not characterized by genomic instability as tumor cells are [30–31]. Fourth, inducing tumor-associated macrophages to acquire an M1-phenotype might be a novel immunotherapy strategy [32] and cysmethynil could be used for this purpose though capable to target only pleural and not tumoral macrophages. Fifth, accumulating data outline the role of Rho and Ras in chemosensitivity of tumor cells [33–35]. Therefore, cysmethynil may display synergy with standard cytotoxic agents, a possibility that should be tested in future studies.

In conclusion, we here demonstrated that cysmethynil curtails experiential MPE formation, irrespective of its *in vivo* activity on the growth of the pleural tumors. Its effects are mostly related to inhibition of new vessel formation and vascular hyper-permeability and to an increase of M1-macrophage homing. The clinical development and testing of cysmethynil as a palliative pharmacological intervention for patients with MPE might be therefore worthy of pursuit.

## MATERIALS AND METHODS

### Tumor cell lines

Murine lung adenocarcinoma cells (Lewis Lung Cells, LLC) were purchased from American Type Collection Cultures (Manassas, VA). AE17 murine mesothelioma cells [18] were generated by Dr B. Robinson and kindly provided by Dr YCG Lee, Perth, Western Australia. Cells were maintained in Dulbecco's Modified Eagle Medium (containing 10% heat-inactivated FCS, L-glutamine and 100 mg/l penicillin and streptomycin (Gibco, Grand Island, NY). Both cell lines used in the present study were periodically monitored for mycoplasma presence. Their morphology was examined on a regular basis. The effects of the inhibitor in tumor cell viability were assessed using the MTS (3-[4,5-dimethylthiazol-2-yl]-5-[3-carboxymethoxyphenyl]-2-[4-sulphophenyl]-2H-tetrazolium, inner salt) (Promega, Madison, WI) assay.

### Animal studies

Mice were purchased form BSRC Al. Fleming (Vari, Greece) or Hellenic Pasteur Institute (Athens, Greece) and were housed at the Animal Model Research Unit of Evangelismos Hospital, (Athens, Greece). Experiments were approved by the Veterinary Administration Bureau, Prefecture of Athens, Greece under compliance to the national law and the EU Directives. Adenocarcinoma LLC (1.5 × 10^5^) or mesothelioma AE17 (5 × 10^5^) cells were intrapleurally injected to 8–10 week-old male, C57BL/6 syngeneic mice and cysmethynil (purchased from Duke University, Durham, NC) was intraperitoneally administered at 0.2 mg/g three times weekly starting at the 4th day of tumor cell injection since pleural tumors are already evident at this time point. This regimen was previously found to be effective in murine models of PC3 prostate cancer cells [19] and HepG2 hepatocellular carcinoma cells [15] and in our hands did not affect mouse welfare status when tested in preliminary experiments. Animals were euthanized 13 days post injection at this point usually exhibiting distress and impaired motility. Pleural fluid was retrieved and volume was measured. To determine pleural tumor burden, the thoracic cavity was opened and all of the mesothelioma masses were carefully excised (until achieving “clean” pleural surfaces) and weighed or lungs were harvested in order for the adenocarcinoma visceral pleural foci to be counted under a stereoscope (Stemi DV4, Zeiss, Germany).

### Histology and flow cytometry

Formalin-fixed, paraffin-embedded tumor tissue sections were stained with anti-Proliferating Cell Nuclear Antigen (PCNA) (Santa Cruz Biotechnology, CA), or anti-CD31 (Abcam, Cambridge, MA) as previously described [20]. Tumor cell apoptosis was evaluated by TUNEL assay (Roche, Penzberg, Germany) [20]. For flow cytometry analysis, pleural fluid and tumor cells were fixed, permeabilised and stained with anti-CD45-APC-Cy-7 (pan-leucocyte marker), anti-CD11b-FITC (mature myeloid cell marker), anti-F4/80-PerCP (tumor-associated macrophages), anti-IL10-PE (prototype cytokine of pro-tumor M2 phenotype) and anti-IL12-APC (prototype cytokine of anti-tumor M1 phenotype). The M1/M2 ratio was used as a marker of macrophage polarization. Samples were analyzed using the multicolor flow cytometer BD FacsCanto II. Flow cytometric data were analyzed using the BD FACSDiva Version 6.1.3 Software (BD Bioscience, Athens, Greece). Inflammatory cells were selected based on their forward and side scatter profiles and their CD45 positive staining.

### *In vivo* vascular permeability assay

In order to assess pleural vascular permeability, MPE-bearing animals were intravenously injected with 200 μl, of the albumin-binding Evans blue dye (4 mg/ml) upon anesthesia and sacrificed 1 hour later. Pleural fluid and blood were collected, centrifuged and supernatants' Evans blue levels were determined by measurement at 630 nm. Concentration of the dye was interpolated using a standard curve and permeability was evaluated by the ratio of pleural fluid/serum Evans blue concentration. This ratio expresses the degree of albumin leakage from the circulation to the pleural space through a leaky pleural vasculature.

### *In vitro* macrophage polarization and migration assays

Bone marrow-derived macrophages from C57BL/6 mice were generated using standard techniques [21] and were seeded at 1 × 10^5^ cells/1,5 ml in the lower compartments of (0.4 μm) Transwell system with an equal population of LLC or AE17 cells loaded on the upper chambers. Macrophages were treated with 20 μM (non-toxic dose) cysmethynil or vehicle 24 h later and left for another 24 h. At the end of the incubation time, macrophages were collected, fixed, permeabilised and stained for CD11b, F4/80 and IL-10, IL-12 presence for flow cytometric analysis.

For migration studies macrophages were serum starved overnight and treated with vehicle or 20 μM cysmethynil. Two hours later, cells were collected and loaded onto the upper compartments of Transwells (8-μm pore size), while the day before lower compartments were seeded with 3 × 10^4^ LLC or AE17 cells. All compartments were serum deprived. Migrated cells were fixed, stained and counted under a microscope.

### *In vitro* endothelial cell viability, migration and tube formation

Human Umbilical Vein Endothelial Cells (HUVEC) were isolated as previously described [23] and maintained in M199 full medium (15% FCS, 200 μg/ml ECGS and 5U/ml heparin). HUVECs were used until passage 2. HUVEC viability was assessed using the MTS assay. For migration studies HUVECs were serum starved overnight, treated with vehicle or 5 μM (non-toxic dose) cysmethynil and loaded on the upper compartments of Transwells at 1 × 10^5^ cells/chamber. Lower chambers were filled with full medium and 6 hours later, migrated cells were fixed, stained and counted under a microscope.

For endothelial cell tube formation, 24-well plates were coated with 40 μl Matrigel (growth factor reduced, BD Bioscience, Athens, Greece) and incubated for 1 h at 37°C. Serum deprived HUVEC were loaded onto pre- coated wells at a density of 4 × 10^4^ cells/well and incubated for 18 h in the presence of cysmethynil concentrations that do not affect cell viability (0.5–20 μM) or vehicle. Following incubation tubes were fixed, stained and tube network was imaged at 200X magnification under a phase contrast microscope. Images were subsequently analyzed for network length and branching points using ImageJ (NIH, Bethesda, MD).

### Statistics

All values are presented as mean ± standard error of mean (SEM). Differences between groups were evaluated using the Student's *t*-test or the one-way ANOVA with least square difference post-hoc tests, as appropriate. *P* values < 0.05 were considered significant. Statistical analysis was performed using the Statistical Package for the Social Sciences v.13.0.0 (IMB, Armonk, NY).
